# Effect of Surfactants on Longevity of Submerged Superhydrophobic Surfaces

**DOI:** 10.1021/acs.langmuir.5c05968

**Published:** 2025-12-24

**Authors:** Ankit Gupta, Hangjian Ling

**Affiliations:** Department of Mechanical Engineering, 14709 University of Massachusetts Dartmouth, Dartmouth, Massachusetts 02747, United States

## Abstract

The gas trapped on a submerged superhydrophobic surface (SHS) could diffuse into the surrounding liquid, reducing the SHS longevity. Although surfactants are known to inhibit mass transfer between gas and liquid phases, their influence on SHS longevity remains unclear. In this work, we experimentally investigate the effect of surfactants on the mass transfer from the SHS to the surrounding liquid and the resulting SHS longevity. Four surfactants, 1-pentanol, Triton X-100, 2-propanol, and methanol, were tested over a range of concentrations and on three types of SHS texture geometries: microholes, microposts, and a randomly roughed texture. We found that at sufficiently high surfactant concentrations, the SHS longevity decreased significantly due to a sudden wetting transition triggered probably by surface energy minimization. In contrast, at low surfactant concentrations, the SHS longevity was larger than that in pure water and increased with an increasing surfactant concentration. This extended SHS longevity was observed for all four surfactants and across all three texture geometries. Our results demonstrate the universal role of surfactants in reducing the mass transfer coefficient and highlight the importance of accounting for surfactants when assessing the SHS longevity in real-world applications.

## Graphical abstract

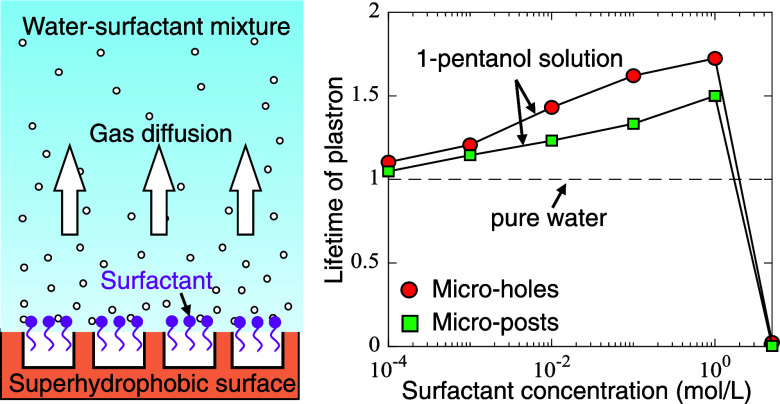

## Introduction

When submerged in a liquid, a superhydrophobic surface (SHS) traps a thin layer of gas (or plastron) within the surface textures, forming a Cassie–Baxter state. The presence of gas on SHS has promoted many desired functions, including reducing friction drag,^1,2^ enhancing heat and mass transfer,^3^ controlling bubble formation,^4,5^ protecting underwater surfaces from biofouling,^6^ icing,^7^ and corrosion.^8^ However, one challenge that limits the broad application of SHS is the gas diffusion issue: the gas trapped on the SHS may diffuse into the ambient liquid, leading to a wetting transition to the Wenzel state. Understanding this diffusion process is crucial for the design and manufacturing of long-lasting SHS.^9^ Furthermore, in many real-world systems, such as marine environments, surfactants are often present in the liquid, accumulate at the gas–liquid interface, and affect the mass transfer between liquid and gas phases.^10,11^ However, past studies have mainly focused on the longevity of SHS in pure water.^12^ It remains unclear whether and how surfactants affect the gas transfer from the SHS to liquid and subsequently the SHS longevity. This work aims to address this knowledge gap by experimentally measuring the longevity of SHS in liquids contaminated by surfactants.

The longevity of SHS in pure water has been extensively investigated in the past few decades. Gas transfer from the SHS to the liquid is primarily because of a difference between the dissolved gas concentration in the bulk liquid (*c*_∞_) and the concentration at the gas–liquid interface (*c*_i_). According to Fick’s first law, the rate of gas transfer is proportional to the local concentration gradient near the interface. The SHS longevity depends on both texture geometries and environmental conditions, such as flow and pressure. Increasing pressure in the liquid^13–15^ or increasing the immersing depth of the sample^16–18^ has been shown to cause an increase of *c*_i_ according to the Henry’s law, and consequently a reduction of SHS longevity. Decreasing *c*_∞_ could also result in a reduction of SHS longevity.^19–21^ Introducing flow over SHS reduces the longevity due to the formation of a thin boundary layer, across which the gas concentration drops sharply from *c*_i_ to *c*_∞_.^22,23^ With increasing flow velocity, the boundary layer thickness decreases, leading to a larger mass flux and a shorter SHS longevity.^24,25^ The enhancement of gas transfer due to flow has been quantified by the dimensionless Sherwood number.^22,26–28^ Other studies^29–34^ have investigated the effects of surface chemistry and surface texture on the SHS longevity. For example, Bourgoun and Ling^31^ numerically showed that increasing texture height and gas fraction (i.e., ratio of gas–liquid contact area to total surface area) leads to an increase of SHS longevity. Arunachalam et al.^35^ found that doubly re-entrant cavities could either extend or reduce the SHS longevity depending on the hydrostatic pressures. Gupta and Ling^36^ found that the SHS longevity could be extended by using a gas soluble and gas permeable material.

However, the longevity of SHS in complex liquids, such as those contaminated by surfactants, has received limited attention. Surfactants are well-known to accumulate at the gas–liquid interface due to their amphiphilic nature. In addition to reducing surface tension,^37,38^ the accumulation of surfactants inhibits mass transfer.^10^ Numerous studies have focused on the effect of surfactants on the mass transfer from rising bubbles to surrounding liquid^39–43^ and across plane interfaces.^44–48^ They showed that the mass transfer coefficient depends on both the surfactant concentration (*c*_s_) and the molecular structure of the surfactant, such as the hydrophobic chain length.^49^ According to the Langmuir adsorption model,^50^ with increasing *c*_s_, the concentration of the surfactant at the interface initially increases and then saturates when *c*_s_ reaches the critical micellar concentration (CMC). The mass transfer coefficient was found to follow the same trend: with increasing *c*_s_, it initially decreases and then becomes nearly constant beyond CMC.^41^ Recently, Awashra et al.^32^ examined the longevity of SHS in biofluids (mixtures of water with bovine serum albumin, fetal bovine serum, and glucose). They found that biofluids reduced the SHS longevity due to the biofluids lower surface tension and biomolecular adsorption. To the best of our knowledge, whether a surfactant can reduce the rate of gas transfer from SHS to the liquid and consequently extend the SHS longevity has not yet been fully investigated.

Although there is limited work on the effect of surfactants on the SHS longevity, the effect of surfactants on the wetting behavior of a droplet seating on SHS has been well investigated.^51^ Mohammadi et al.^52^ found that the contact angle reduced with increasing the surfactant concentration in the droplet. They showed that at high surfactant concentration, where the intrinsic contact angle on a smooth surface of identical material was below 90°, the SHS did not turn to superhydrophilic. They hypothesize that surfactant may hinder the penetration of liquid to the microcapillary surface pores due to unfavorable thermodynamic conditions. Later, Chang et al.^53^ showed that with increasing surfactant concentration, SHS remained in the hydrophobic range for linear surfactants (e.g., sodium dodecyl sulfate) but turned to superhydrophilic for branch-tailed surfactants (e.g., didodecyldimethylammonium bromide, Triton X-100) due to the continuous reduction of solid–liquid interfacial tension. Aldhaleai and Tsai^54^ observed a wetting transition from the Cassie–Baxter state to Wenzel state caused by the surfactant adsorption. They proposed a theoretical model for the contact angle of the surfactant-laden droplet by considering surfactant adsorption at the liquid–gas and liquid–solid interfaces. They also calculated the free-energy barrier between the Cassie–Baxter state and the Wenzel state to predict the effect of surfactants on the stability of the Cassie–Baxter state.

This study primarily focuses on the effect of surfactants on the longevity of SHS submerged in a gas undersaturated environment. We experimentally measure the SHS longevity in surfactant solutions by using an optical method. We will show that the SHS longevity in the surfactant solution is larger compared to that in pure water and increases with the increasing surfactant concentration. We will confirm the universal behavior of surfactants: whether involving a microbubble trapped on a SHS or a rising bubble in a liquid column, the accumulation of surfactants at gas–liquid interface reduces the mass transfer coefficient.

## Materials and Methods

We performed experiments using the setup shown in Figure 1a. This setup was used in our previous studies^20,36^ for the investigation of the effects of undersaturation level and gas permeability of polydimethylsiloxane on the SHS longevity. The SHS sample (diameter 12.5 mm) was installed at the top of a tube filled with a liquid. To ensure that the only air present in the systems were these trapped within the surface microtextures, the tube was first completely filled with liquid, and any bubbles adhering to the inner wall were removed. The sample was then gently placed on top of the liquid with its textured side facing downward, and the tube was sealed to isolate the system from the surroundings. The tube length was 270 mm. The hydrostatic pressure on the sample was atmospheric pressure. Thus, the air concentration in the liquid near the SHS corresponded to the one saturated with 1 atm, i.e., *c*_i_ = 0.023 kg/m^3^. The air concentration in the bulk liquid was set to *c*_∞_ = 0.3*c*_i_, achieved by degassing 50 mL of solution in a vacuum chamber at an absolute pressure of 0.2 bar for 2 days before transferring it into the tube. An optical oxygen sensor (FirestingO_2_, PyroScience) was installed at the bottom of the tube to monitor the air concentration throughout the experiments. The measured oxygen concentration was converted to air concentration by assuming a constant oxygen fraction of 21% in air.

**Figure 1:**
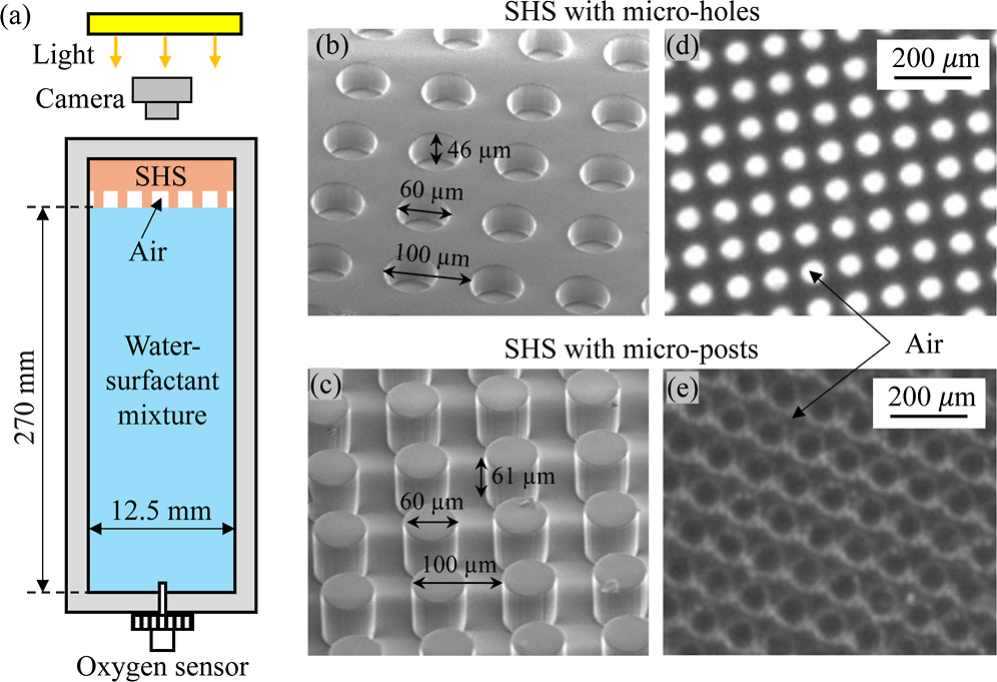
(a) Experimental setups for measuring the longevity of SHS in surfactant solution; (b,c) scanning electron microscopy (SEM) image of SHS with microholes (b) and microposts (c); and (d,e) images obtained by the optical setup shown in (a) for SHS with microholes (d) and microposts (e) at the beginning of the wetting process (bright areas on the images indicate the presence of gas trapped on the SHS).

To measure the wetting transition induced by the gas diffusion, an LED light illuminated the SHS, and the light reflected from the SHS was recorded by a CMOS camera (FLIR Grasshopper 3, pixel size 5.5 mm, 2048 × 2048 pixels). The field of view was 13 × 13 mm, covering the entire SHS surface. Two SHS texture geometries: microholes and microposts, as shown in Figure 1b,c, were mainly used in this study. These textures were created on poly­(dimethylsiloxane) (PDMS) substrates through a standard soft-lithography technique. The microhole texture consisted of cavities with a radius *r* = 30 μm, depth *h* = 46 μm, and wavelength λ = 100 μm. The micropost texture featured pillars with a radius *r* = 30 μm, a height *h* = 61 μm, and a wavelength λ = 100 μm. The thickness of the PDMS surface was 2 mm. Since the PDMS is intrinsically hydrophobic, no additional coatings were applied to modify the surface chemistry. As shown in Figure 1d,e, when submerged in pure water, air (indicated by the bright areas on the images) was trapped within the surface textures, confirming the formation of the Cassie–Baxter state.

The surfactant primarily used in this work was the nonionic, 1-pentanol (Sigma-Aldrich, # 398268, >99%, CAS # 71-41-0). The surfactant solutions were prepared by mixing 1-pentanol with deionized water. We varied the concentration of 1-pentanol in the solution, denoted as *c*_s_, from 0 to 5 mol/L, where *c*_s_ = 0 corresponded to pure water and the highest concentration exceeded the critical micellar concentration (CMC). The reason for choosing 1-pentanol was because of its well-characterized adsorption properties and its short time scale (<0.1 s) for reaching the equilibrium concentration at the interface.^37,55^ According to the references,^1,55,56^ pentanol has a maximum interfacial concentration of Γ_∞_ = 5.90 × 10^–6^ mol/m^2^ and an adsorption coefficient of *K* = 66 L/mol. Under equilibrium conditions, the surface concentration Γ of 1-pentanol can be described by the Langmuir model, expressed as

1 $$\Gamma =\Gamma_{\infty }\;Kc_{s}/\left(1+Kc_{s}\right)$$

As will be shown later, we found that at low surfactant concentrations, the SHS longevity was larger than that in pure water, primarily due to the reduction of mass transfer coefficient caused by the accumulation of 1-pentanol at the interface. To examine whether this trend applies to other surfactants, we performed additional experiments using three other nonionic surfactants: Triton X-100 (Sigma-Aldrich, # X-100, CAS # 9036-19-5), 2-propanol (Mcmaster, # 3190K812, >99%, CAS # 67-63-0), and methanol (Sigma-Aldrich, # 179957, >99.5%, CAS # 67-56-1), at low concentrations varying from 0 to 0.1 mol/L. All solutions were prepared by mixing the respective surfactant with deionized water. These three surfactants were arbitrarily selected from those previously reported to reduce the mass transfer coefficient. For examples, Triton X-100 and methanol were shown by Lebrun et al.^41^ and Mölder et al.,^10^ respectively, to reduce the mass transfer rate. Future studies could select specific types of surfactants to investigate the effects of the surfactant molecular structure such as hydrophobic chain length on SHS longevity.

Figure 2 shows the equilibrium surface tension between air and the surfactant solution, denoted as σ, as a function of *c*_s_. The equilibrium surface tension was measured using the pendant droplet method.^57^ For 1-pentanol at low concentrations (*c*_s_ < 0.1 mol/L), the results showed good agreement with the Szyszkowski model:^56^ σ = σ_0_ – *RT*/ω ln (1 + *Kc*_s_), where σ_0_ is the surface tension of pure water, *R* is the ideal gas constant, *T* is the temperature, and ω is the limiting partial molar area of the solute at the surface (ω = 1.48 × 10^5^ m^2^/mol for 1-pentanol). For 1-pentanol at high concentrations (*c*_s_ > 1 mol/L), σ became nearly constant, suggesting that the CMC and maximum interfacial concentration had been reached. According to Figure 2, the CMC of 1-pentanol is approximately 1 ± 0.5 mol/L, in agreement with previous studies.^58^ According to Lebrun et al.,^41^ the CMC of Triton X-100 is 0.3 × 10^–3^ mol/L. Methanol and 2-propanol do not exhibit CMC behavior as both are completely miscible with water. The chemical formula, CMC values, and concentration range (*c*_s_) of the four surfactants used in this work are summarized in Table 1.

**Figure 2:**
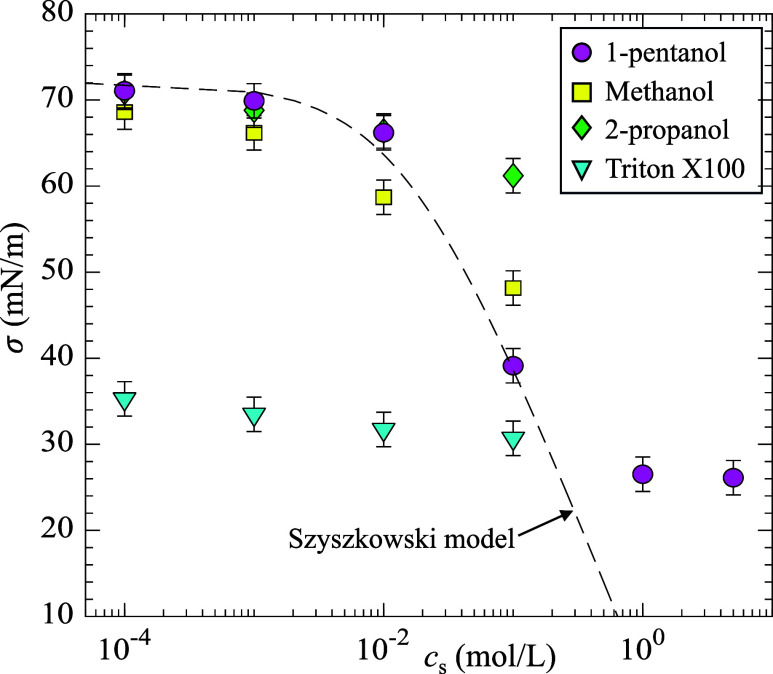
Equilibrium surface tension as a function of the surfactant concentration for four different surfactants used in this work.

**Table 1:** Chemical Formula, Critical Micelle Concentration (CMC), and Range of *c*_s_ of the Four Different Surfactants Used in This Study^a^

surfactant name	chemical formula	CMC (mol/L)	*c*_s_ (mol/L)
1-pentanol	C_5_H_12_O (or CH_3_(CH_2_)_4_OH)	1.0 ± 0.5	1 × 10^–4^ to 5
Triton X-100	C_14_H_22_O(C_2_H_4_O)_*n*_, *n* ≈ 9–10	0.3 × 10^–3^	1 × 10^–4^ to 0.1
methanol	CH_4_O (or CH_3_OH)	-	1 × 10^–4^ to 0.1
2-propanol	C_3_H_8_O (or (CH_3_)_2_CHOH)	-	1 × 10^–4^ to 0.1

a Methanol and 2-propanol are miscible with water and do not have CMC values.

## Results and Discussion

First, we examined the longevity of SHS in pure water (*c*_s_ = 0). Figure 3a,b shows the wetting transitions for SHS with microholes and microposts, respectively. A small 400 pixels × 400 pixels region selected from the original 2048 pixels × 2048 pixels image was shown for clarity. As shown, the image transitioned from bright to dark as the gas slowly dissolved into the water. This occurs because the light reflected from the gas–liquid interface is stronger than that from the solid–liquid interface. For SHS with microholes, the gas was trapped within isolated cavities, creating numerous discrete plastrons. With increasing time (*t*), the number of bright spots (or the number of plastrons) decreased. For SHS with microposts, the gas was trapped in the interconnected spaces between pillars, forming a single continuous plastron. With increasing *t*, the bright region (or the plastron) shrank mainly in the horizontal direction.

**Figure 3:**
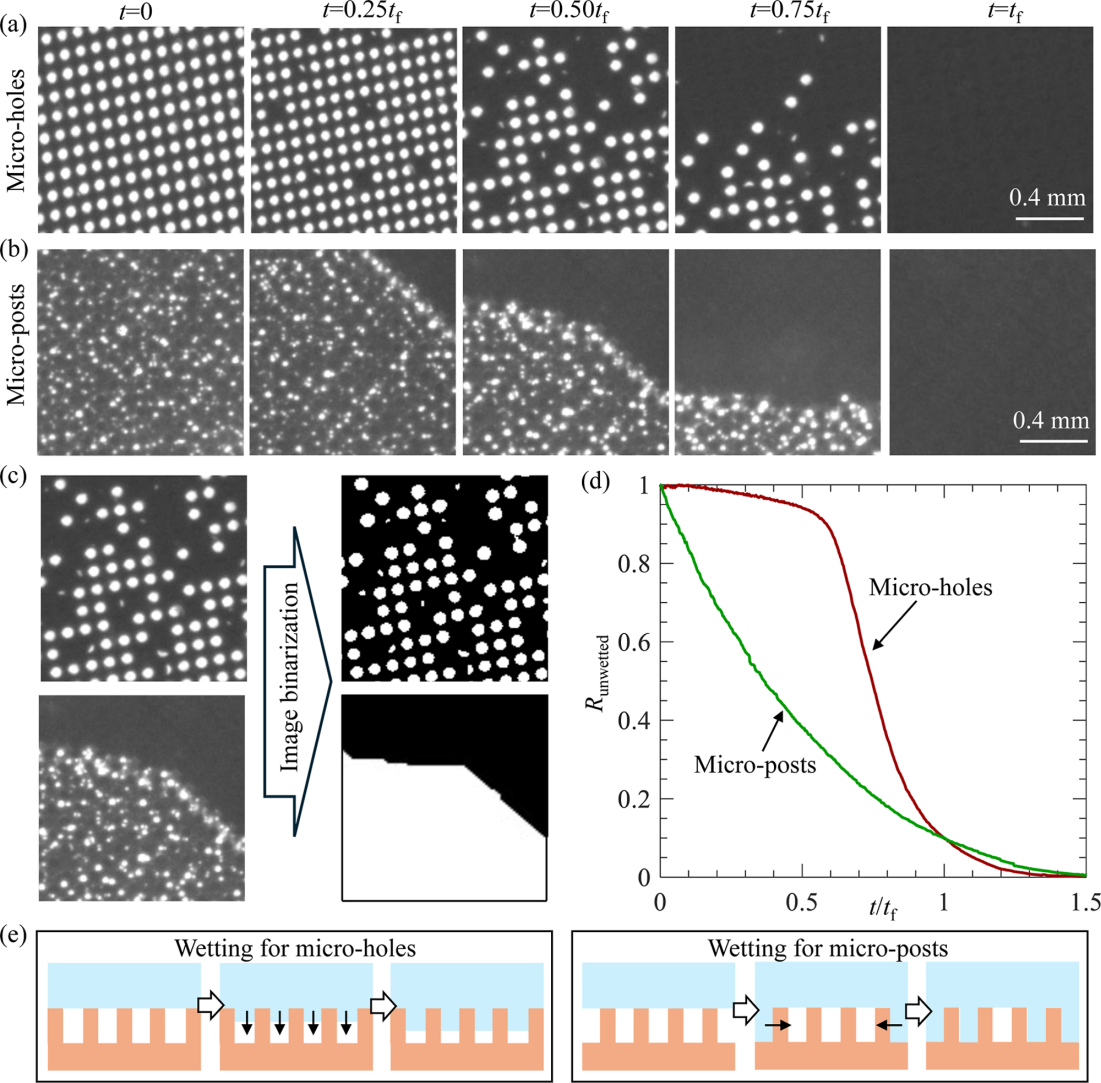
(a,b) Wetting transition for SHS with microholes (a) and microposts (b) in pure water; (c) the same images before and after the binarization; (d) *R*_unwetted_ as a function of *t*/*t*_f_ corresponding to data shown in (a,b); (e) schematics of time evolutions of the gas–liquid interface during the initial wetting process for SHS with microholes and microposts (the arrows indicate the propagating direction of the interface; the interface shape was simplified and may not represent the actual interface). *t*_f_ is defined as the time when *R*_unwetted_ = 0.01.

Following the data analysis procedure established in our previous work,^20^ we calculated the SHS longevity (*t*_f_) from the recorded images. The procedure involved binarization of the raw images based on intensity, as shown in Figure 3c. After binarization, we calculated the ratio of unwetted surface area as *R*_unwetted_, where *R*_unwetted_ = 1 at the beginning of wetting process and *R*_unwetted_ = 0 when all gas was dissolved into the liquid. Figure 3d shows the variation of *R*_unwetted_ as a function of time for microholes and microposts. As expected, with increasing time, *R*_unwetted_ decreased monotonically to 0 for both textures. For microholes, *R*_unwetted_ decreased slowly at first and then dropped sharply. In contrast, for microposts, *R*_unwetted_ decreased at a slower rate. This difference might be caused by different behaviors of the gas–liquid interface during wetting transition, as illustrated in Figure 3e: for microholes, the interface penetrated vertically into the holes; while for microposts, it contracted horizontally parallel to the surface. Finally, SHS longevity was defined as the time at which *R*_unwetted_ decreased to a threshold of 0.01 (note that this threshold was arbitrarily selected, and the conclusions of our work remain valid for other reasonable thresholds).

Next, we examined the longevity for SHS submerged in 1-pentanol solutions with concentrations ranging from 0.0001 to 5 mol/L. Figure 4a,b presents the time variation of *R*_unwetted_ for SHS with microholes and microposts, respectively. Supporting Information Figure S1 shows the time-series images demonstrating the wetting transition for SHS with microholes in 1-pentanol solutions. For low concentrations (*c*_s_ < 1 mol/L), with increasing *c*_s_, *R*_unwetted_ decreased to 0 at slower rates, suggesting that the mass transfer rate was reduced in surfactant solution. Figure 4c shows *t*_f_/*t*_f0_ as a function of *c*_s_. Here, *t*_f0_ is the SHS longevity in pure water. Clearly, for *c*_s_ < 1 mol/L, *t*_f_/*t*_f0_ was larger than 1 and increased with increasing *c*_s_. These results suggest that the accumulation of surfactant at the interface suppresses mass transfer from SHS to liquid, consistent with the effect of surfactants on the gas transfer between a rising bubble and liquid reported in the literature.

**Figure 4:**
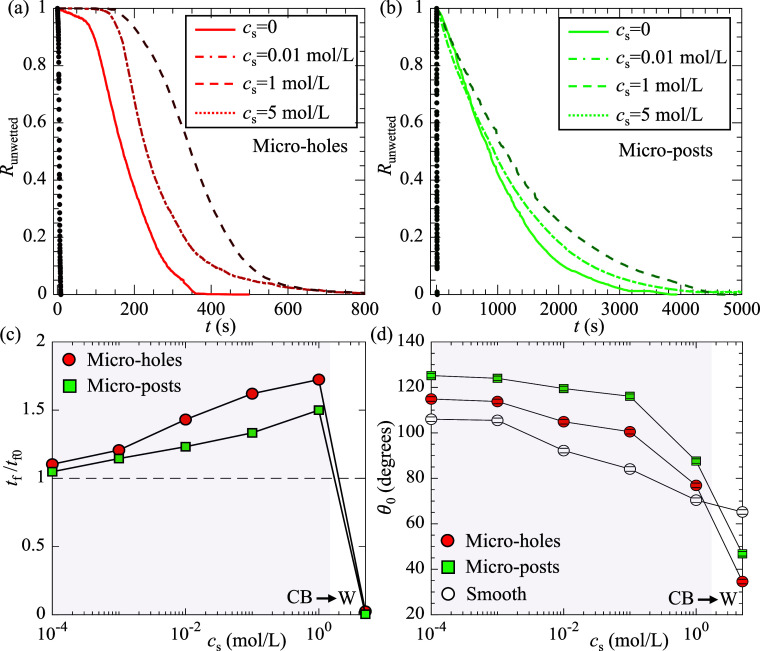
(a,b) *R*_unwetted_ as a function of time at four concentrations (*c*_s_ = 0, 0.01, 1, and 5 mol/L) for SHS with microholes (a) and microposts (b); (c) SHS longevity as a function of surfactant concentrations; and (d) static contact angle as a function of surfactant concentrations measured on textured PDMS surface and smooth PDMS surface. CB and W in (c,d) denote the Cassie–Baxter (CB) state and the Wezel (W) state, respectively.

However, at the highest concentrations *c*_s_ = 5 mol/L, for both texture geometries, *R*_unwetted_ rapidly reduced to 0 with increasing time (Figure 4a,b), and the SHS longevity reduced to nearly 0 (Figure 4c). This is probably because at sufficiently high surfactant concentrations, the Cassie–Baxter state becomes thermodynamically unstable, leading to a wetting transition driven by surface energy minimization.^54^ To verify this, we measured the static contact angle, θ_0_, using the sessile drop method. Figure 4d shows θ_0_ as a function of *c*_s_ for both textured and smooth PDMS surfaces. For *c*_s_ = 5 mol/L, the textured surfaces exhibited smaller θ_0_ than the smooth PDMS surface, suggesting that the droplet was in the Wenzel state. In fact, as shown in Supporting Information Figure S2, at *c*_s_ = 5 mol/L, the measured static contact angle agreed well with the prediction by the Wenzel equation: cos θ_W_ = *r*_w_ cos θ_Y_, where *r*_w_ is the Wenzel roughness (the ratio of total surface area to the projected surface area) and θ_Y_ is Young’s contact angle on a smooth surface of the same material. In contrast, the Cassie–Baxter equation: cos θ_CB_ = ϕ_s_ cosθ_Y_ – 1 + ϕ_s_, where ϕ_s_ is the solid fraction, overpredicted the measured contact angles. A droplet switching from the Cassie–Baxter to the Wenzel state with increasing *c*_s_ was also observed by Aldhaleai and Tsai.^54^ The observation that θ_0_ continuously decreased with increasing *c*_s_ agrees with the findings of Chang et al.^53^ and suggests that both liquid–solid and liquid–vapor interfacial tensions decrease due to surfactant accumulation.

To confirm that at low surfactant concentrations, the wetting transition was mainly caused by gas diffusion, we compared our experimental results to a one-dimensional (1D) gas diffusion model, as illustrated in Figure 5a. In this model, a thin layer of gas with height *h*_1D_ is in contact with an undersaturated liquid occupying a semi-infinite domain. The initial gas concentration in the liquid is prescribed as *c*_∞_, while the gas concentration at the gas–liquid interface is maintained at a constant value *c*_i_ (*c*_i_ > *c*_∞_). The diffusion of gas from the thin gas layer to the liquid is governed by the following equation

2 $$\frac{\partial c}{\partial t}=D_{g}\frac{\partial^{2}c}{\partial y^{2}}$$

where *c* is the gas concentration in the liquid, *D*_g_ is the diffusion coefficient of gas in the liquid (here, for the diffusion of air in water at room temperature, *D*_g_ = 2.0 × 10^–9^ m^2^/s), *t* is time, and *y* is the vertical distance from the gas–liquid interface (with *y* = 0 denoting the location of the interface).

**Figure 5:**
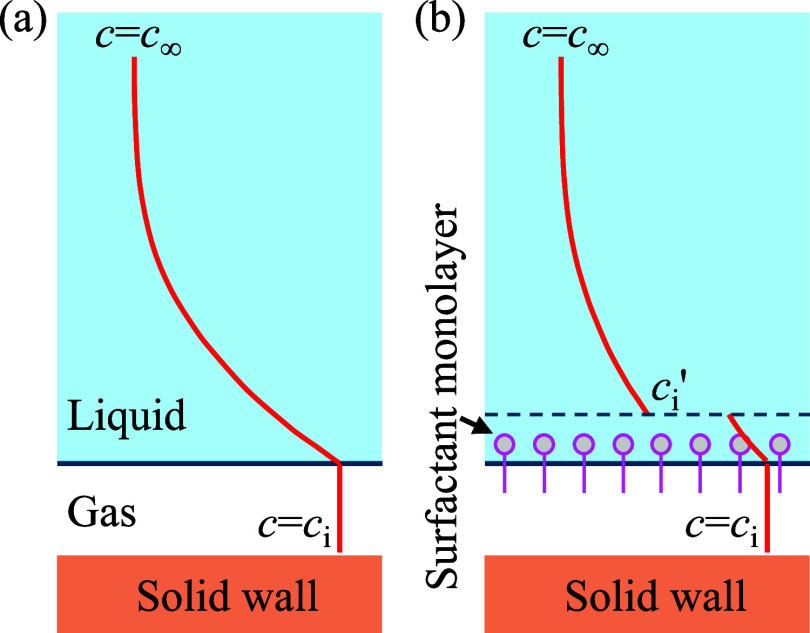
(a,b) Illustration of the mechanism of the reduced mass transfer coefficient due to the barrier effect of surfactants. Schematic diagrams show the gas concentration profile at a given time instant during the diffusion process in (a) pure water and (b) surfactant solution. Gas diffusion is a transient problem, and the concentration profile evolves over time as the gas dissolves into the liquid. The concentration profile in (b) is adapted with permission from.^44^ Copyright [1988] Elsevier.

Assuming a fixed gas–liquid interface at *y* = 0 and solving the 1D diffusion equation (see Supporting Information), we found the time required for all gas within the thin layer to be dissolved as

3 $$t_{f}=\frac{\pi }{4}\frac{{\rho_{g}}^{2}h_{1D}^{2}}{D_{g}{\left(c_{i}-c_{\infty }\right)}^{2}}$$

where ρ_g_ is the density of gas (here for air, ρ_g_ = 1.2 kg/m^3^). Considering that a SHS with texture height *h* and gas fraction ϕ_g_ has the same amount of trapped gas to a 1D gas layer of effective thickness of *h*_1D_ = *h*ϕ_g_, we estimated the time required for all gas on the SHS to be dissolved as

4 $$t_{f}=\frac{\pi }{4}\frac{{\rho_{g}}^{2}h^{2}\phi_{g}^{2}}{D_{g}{\left(c_{i}-c_{\infty }\right)}^{2}}$$

By substitution the experimental parameters into eq 4, the 1D diffusion model predicts *t*_f_ = 4176 and 369 s for SHS with microposts and microholes, respectively, as listed in Table 2. These predicted values are in good agreement with the experimentally measured longevity *t*_f_ = 3550 and 350 s for the corresponding textures in pure water. Therefore, we confirm that, in pure water and at low surfactant concentrations, the wetting transition of SHS is primarily governed by gas diffusion.

**Table 2:** Comparison between the Experimentally Measured and Theoretically Predicted SHS Longevity in Pure Water

sample	texture height (μm)	gas fraction	measured *t*_f_ (s)	predicted *t*_f_ (s)	error in *t*_f_ %
microposts	61	0.72	3550	4176	15
microholes	46	0.28	350	369	5

The phenomenon in which the accumulation of surfactants at the gas–liquid interface reduces the mass transfer coefficient is known as the barrier effect,^44,48^ as illustrated in Figure 5b. Surfactant molecules concentrate at the interface to form a monolayer that imposes resistance to gas transport across it. This resistance can be modeled by a sudden drop in the gas concentration across the monolayer.^48^ Consequently, the concentration gradient immediately above the interface decreases, leading to a decrease in mass flux. The value of interfacial resistance is related to the molecular structure of the surfactant, such as the polarity and molecular weight of the hydrophilic group^47^ and the length of the hydrophobic chain.^45,46^ One can model the effect of surfactants on mass transfer and SHS longevity by prescribing a diffusion coefficient within the monolayer, a monolayer thickness, and a gas concentration in liquid just above the monolayer *c*_i_’. The development of such a detailed model is left for future work.

To confirm that the wetting transition at sufficiently high surfactant concentrations was caused by the instability of the Cassie–Baxter state, we calculated the critical contact angle (θ_cr_) for the sudden Cassie–Baxter to Wenzel state transition using the Lafuma and Quere equation :^59^

5 $$\cos\theta_{cr}=\left(\phi_{s}-1\right)/\left(r_{w}-\phi_{s}\right)$$

When the Young’s contact angle of the smooth surface with the same material (θ_0_) is greater than θ_cr_, the Cassie–Baxter state is more thermodynamically stable than the Wenzel state. Based on the given texture parameters, we found θ_cr_ = 90.5° and 91.4° for SHS with microholes and microposts, respectively. As shown in Figure 4d, at low surfactant concentrations (*c*_s_ < 0.01 mol/L), θ_0_>θ_cr_, confirming that the Cassie–Baxter state was stable. However, for *c*_s_ > 1 mol/L, θ_0_ was less than θ_cr_, suggesting that the Cassie–Baxter state became thermodynamically unstable.

Moreover, we examined whether other types of nonionic surfactants have effects similar to 1-pentanol, i.e., extending the SHS longevity when the concentration is relatively low. To this end, we prepared solutions for Triton X-100, 2-propanol, and methanol and varied the surfactant concentration from 0 to 0.1 mol/L. Since the effect of surfactants on SHS longevity was consistent between microholes and microposts (as shown in Figure 4), we performed experiments only on microholes. Figure 6a–c shows the time evolution of *R*_unwetted_ at different surfactant concentrations for Triton X-100, 2-propanol, and methanol, respectively. Figure 6d presents *t*_f_/*t*_f0_ as a function of *c*_s_. Clearly, regardless of the surfactant type, SHS longevity increased with increasing *c*_s_, demonstrating the universal gas-transfer inhibiting effect of surfactants. Among the tested surfactants, Triton X-100 produced the largest enhancement in SHS longevity at the same concentration. The observed differences in SHS longevity among surfactants probably stem from variations in absorption dynamics and molecular structures, such as hydrophobic chain length. A detailed investigation of the effect of the nature of surfactants on SHS longevity is left for future work. Although only nonionic surfactants were tested in this work, we expect that ionic surfactants have similar effects as both nonionic and ionic surfactants have been previously shown to reduce the mass transfer coefficient.^41^

**Figure 6:**
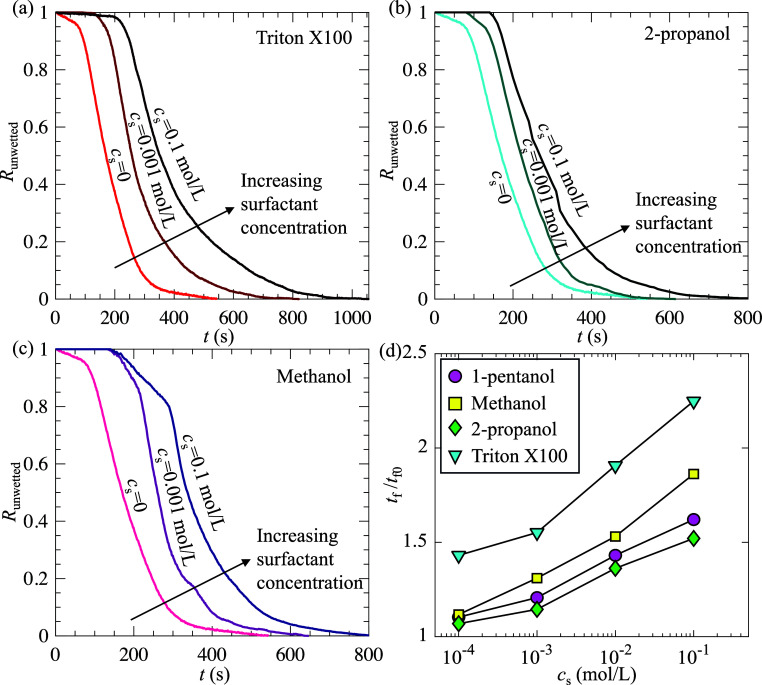
(a–c) *R*_unwetted_ as a function of time at three surfactant concentrations (*c*_s_ = 0, 0.001, and 0.1 mol/L) of Triton X-100 (a), 2-propanol (b), and methanol (c); and (d) SHS longevity as a function of surfactant concentration. The data shown are for SHS with microholes.

To confirm that the extended SHS longevity in surfactant solution was primarily due to the reduction of the mass transfer coefficient, we calculated the liquid-side time-averaged mass transfer coefficient *k*_ave_ over the period 0 < *t* < *t*_f_ using the following equation

6 $$k_{ave}=m_{0}/\left[A_{ave}\left(c_{i}-c_{\infty }\right)t_{f}\right]$$

where *m*_0_ (kg) is the initial mass of gas trapped on the SHS, *A*_ave_ (m^2^) is the time-averaged surface area covered by the gas (projected area normal to the SHS), and *c*_∞_ and *c*_i_ are the gas concentrations in the bulk liquid and at the interface, respectively. For simplification, eq 6 assumes that all gases initially trapped on the SHS completely dissolved into the liquid during the wetting process. However, due to the complex interface shape and possible interface instabilities, full dissolution is not always required for transition to the fully wetted Wenzel state.^13^ Therefore, eq 6 may slightly overestimate the actual mass transfer coefficient. Based on the temporal evolution of *R*_unwetted_ shown in Figure 3d, *A*_ave_ = 0.68*A*_0_ for microholes and *A*_ave_ = 0.51*A*_0_ for the microposts, where *A*_0_ is the initial surface area covered by the gas. The initial gas mass is given by *m*_0_ = ρ_g_ *A*_0_ *h*, where *h* is the texture height, *A*_0_ *h* is the initial gas volume within the textures. Figure 7a,b shows the variation of *k*_ave_ as a function of *c*_s_ for four different surfactants at relatively low concentrations. Results for higher concentrations, where the Cassie–Baxter state was not stable, are not shown. As expected, for all surfactants, *k*_ave_ decreased with increasing *c*_s_, consistent with the trend observed for the mass transfer from a rising bubble to surfactant solutions.^41^

**Figure 7:**
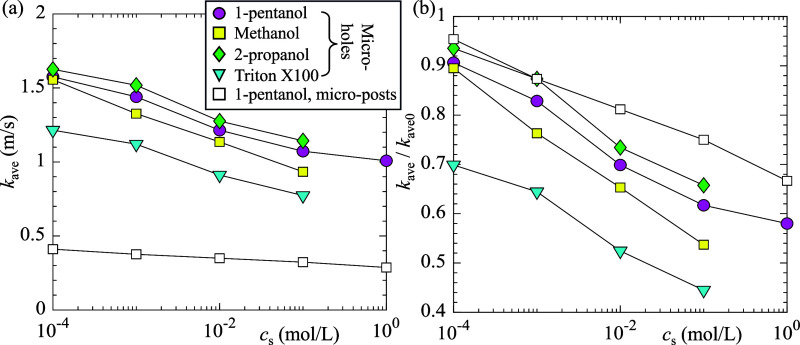
(a,b) Time-averaged mass transfer coefficient of gas from SHS to liquid as a function of surfactant concentrations. The mass transfer coefficients shown in (b) are normalized by the time-averaged mass transfer coefficients obtained in pure water.

Since the CMC values of 1-pentanol and Triton X-100 are known (Table 1), we plotted the SHS longevity and the average mass transfer coefficient as a function of normalized surfactant concentration (*c*_s_/CMC). The results are shown in Supporting Information Figure S3. After normalization, although the two profiles did not perfectly overlap, they exhibited a similar overall trend. According to the literature, when *c*_s_ > CMC, surfactants reach the maximum surface concentration, and further increasing *c*_s_ will not cause a change in the mass transfer coefficient. However, in our experiments, *k*_ave_ for Triton X-100 continued to decrease even when *c*_s_ exceeded the CMC. This discrepancy may arise from the approximations inherent in using the simplified model in eq 6 to estimate *k*_ave_.

Sardeing et al.^50^ proposed an empirical model for the mass transfer coefficient in surfactant solutions expressed as *k*_L_ = *S*_e_ *k*_L1_ + (1 – *S*_e_)*k*_L0_, where *S*_e_ = Γ/Γ_∞_ is surface coverage ratio by surfactant (*S*_e_ = 0 and 1 correspond to pure water and surfactant solution at saturation, respectively), and *k*_L0_ and k_L1_ are the mass transfer coefficients at *S*_e_ = 0 and 1, respectively. This model implies that the mass transfer coefficient is inversely proportional to *S*_e_, consistent with the trends reported in other studies.^47^ We calculated *S*_e_ for 1-pentanol solutions based on eq 1 and compared the experimental results to this model. The results are shown in Supporting Information Figure S4. As shown, the experimental data show reasonable agreement with the model, supporting the interpretation that surfactant accumulation at the interface governs the observed reduction in the mass transfer coefficient.

To investigate whether the surfactant caused a change in the interface shape on SHS, we performed an experiment as illustrated in Figure 8. A SHS consisting of microgrooves (groove width 50 μm, depth 60 μm, and pitch 100 μm), as shown in Figure 8a, was fabricated and submerged in a pressure tank filled with 1-pentanol solution. A camera viewing the sample from the side was used to measure the interface shape, as shown in Figure 8b. Microgrooves were selected instead of microposts and microholes because they allowed the visualization of interface shape using this simple optical configuration. Figure 8c shows the interface shapes when they penetrated into the grooves under pressure at three different *c*_s_. Clearly, with increasing *c*_s_, the interface curvature decreased. To quantify this change, we measured the local advancing contact angle on the side wall of grooves θ_L,adv_, as defined in Figure 8c. As shown in Figure 8d, θ_L,adv_ decreased from 114° to 90° as *c*_s_ increased from 0 to 1 mol/L. We suspect that similar variations in interface shape and θ_L,adv_ occur for SHS with microposts and microholes, as θ_L,adv_ primarily depends on the surface chemistry. Changes in interface shape may also contribute to the extended SHS longevity observed in surfactant solution as they may alter the total amount of gas that must dissolve before the transition to the Wenzel state. Future studies involving direct measurements of the time-evolution of the gas–liquid interface during the wetting process are needed to decouple the relative contributions of the reduced mass transfer coefficient and modified interface shape to SHS longevity.

**Figure 8:**
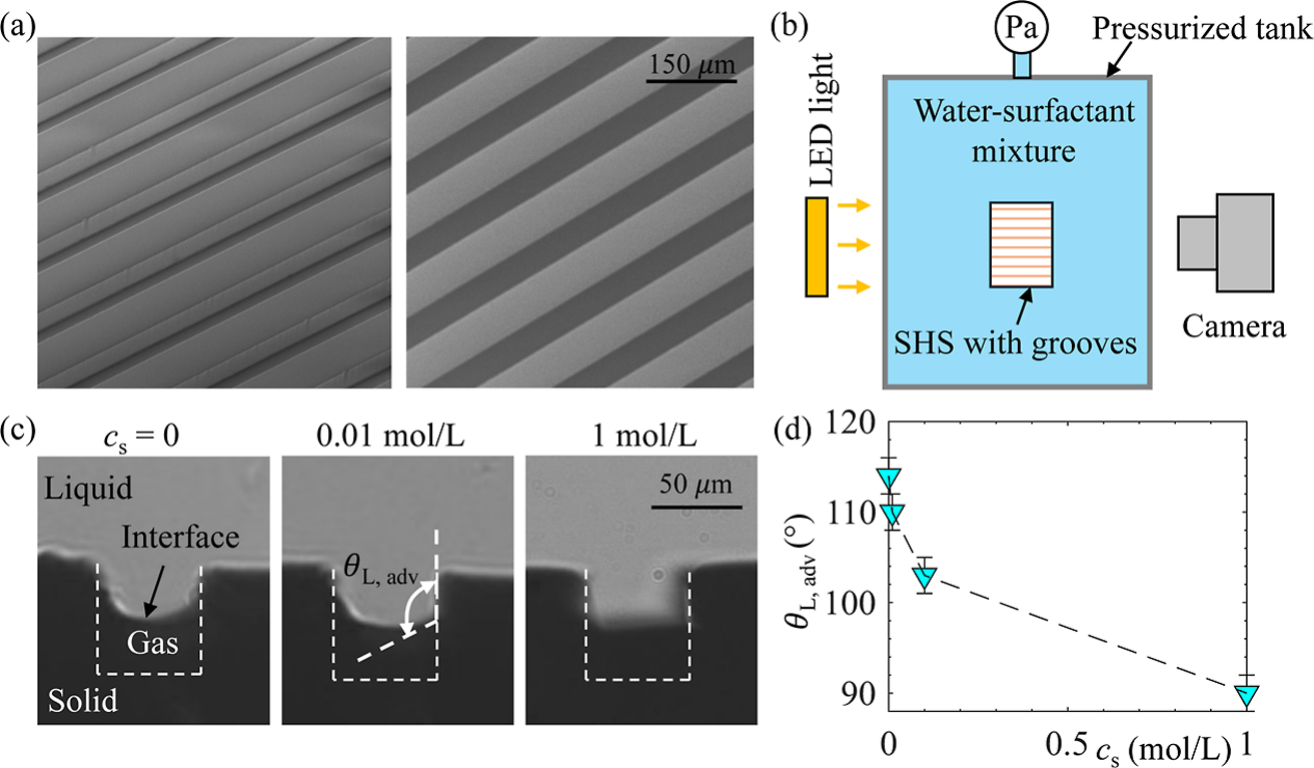
(a) SEM images of the SHS consisting of microridges and microgrooves; (b) experimental setup for measuring the gas–liquid interface using this SHS; (c) images captured by setup in (b) showing the shape of interface when it penetrates into the grooves; and (d) local advancing contact angle as a function of surfactant concentration. The surfactant used here is 1-pentanol.

Lastly, we examined whether the extended SHS longevity observed in surfactant solution on regular textures also applies to randomly textured surfaces. Following our previous work,^60^ we fabricated a SHS with random textures by coating a layer of hydrophobic nanoparticles (Glaco Mirror Coat Zero, SOFT99 Corp) onto a sandpaper with a grit size of 1000. The resulting SHS had a root-mean-square roughness height of 4.7 μm and a static water contact angle of 157°. Figure 9a shows an SEM image of the surface, along with a water droplet resting on it. Figure 9b shows the experimental setup for measuring the longevity of the gas layer on the SHS in water–surfactant solution. This optical setup was based on the principle of total-internal reflection and differed from the one shown in Figure 1a. The alternative setup was necessary because the SHS fabricated with random textures was opaque, whereas the system in Figure 1a required transparent samples. The solution had an air undersaturation level of *c*_∞_ = 0.3*c*_i_, identical to that used for SHS with microholes and microposts. The surfactant used was 1-pentanol at a concentration of *c*_s_ = 0.1 mol/L. Figure 9c shows the wetting process of the SHS in a pure water. With increasing time and gas being dissolved into the liquid, the intensity recorded on the image decreased. From these images, we calculated the *R*_unwetted_. As shown in Figure 9d, *R*_unwetted_ decreased more slowly in surfactant solution (*c*_s_ = 0.1 mol/L) than in pure water (*c*_s_ = 0). This result confirms that the extended SHS longevity in surfactant solution also applies to randomly textured surfaces.

**Figure 9:**
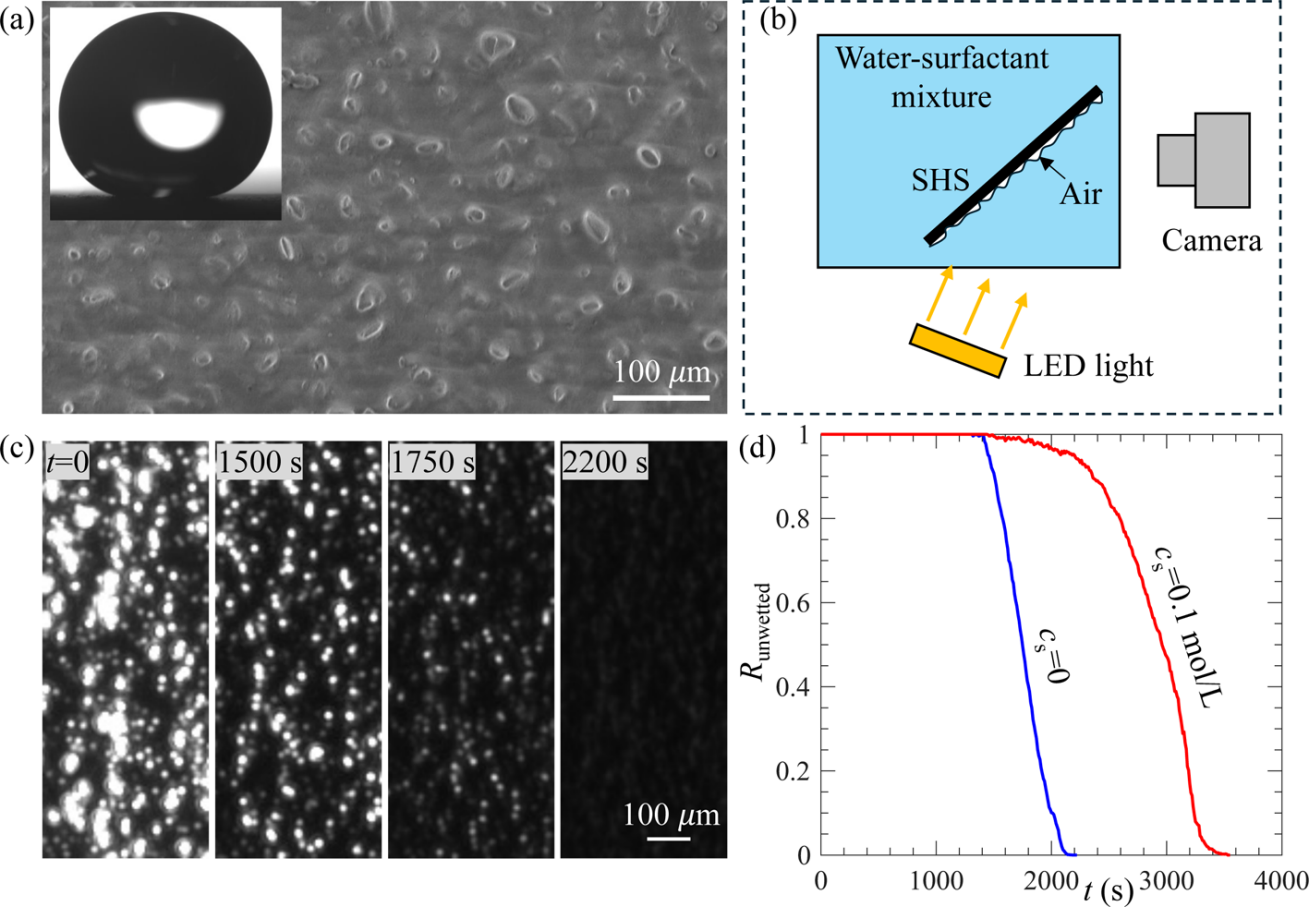
(a) SEM image of SHS with random texture; (b) experimental setup for measuring the longevity of SHS in the water–surfactant mixture; (c) time-series images showing the wetting process induced by gas diffusion in pure water; and (d) *R*_unwetted_ as a function of *t* for *c*_s_ = 0 and 0.1 mol/L.

So far, we have tested SHS with various types of microscale textures. However, for nanoscale textures, the effect of surfactants on the SHS longevity might be different as explained below. For a gas–liquid interface penetrating a groove of width *w*, due to the deformed interface, the gas pressure in the plastron is reduced to *P*_g_ = *P*_w_ – 2σ|cos θ_L,adv_|/*w*, where *P*_w_ is the pressure in liquid near the interface, θ_L,adv_ is the local advancing contact angle, and σ is the surface tension. Consequently, the gas concentration near the interface decreases according to Henry’s law, leading to a smaller concentration gradient and reduced mass flux. In fact, nanoscale bubbles are known to persist in liquid for a long duration because of the surface tension effect.^61^ However, in surfactant solution, due to the reduced surface tension and contact angle, the penetration of interface into the nanotexture may not lead to a significant reduction in gas pressure. As a result, the concentration gradient and mass flux may not be notably affected. Due to the limitations of this work, we leave the study of nanoscale textures for future studies.

All experiments in the current work were performed in stationary liquid. Under flow conditions, the SHS longevity typically decreases compared to that in a stationary liquid due to the advection of gas. Moreover, we expect that the SHS longevity in flows of surfactant-containing liquid may be larger than that in flows of pure water at the same Reynolds number, as explained below. In pure water, the SHS provides a slip boundary condition owing to the nearly shear-free gas–liquid interface and reduces the friction drag.^62^ However, in surfactant solution, this slip boundary could be modified to a no-slip interface due to the gradient of surfactant concentration on the interface in flow direction.^63,64^ Previous studies have shown that a no-slip boundary has lower heat and mass transfer coefficients than a slip boundary.^23,65^ Therefore, SHS longevity in surfactant-laden flows may be larger than that in pure water flows. Future studies are needed to examine the influence of surfactants on SHS longevity under flow conditions and test this hypothesis.

## Conclusion

In summary, we experimentally investigated the effect of surfactants on the mass transfer from the SHS to surrounding liquid and the resulting SHS longevity. Three textured geometries (microholes, microposts, and randomly roughed texture) and four nonionic surfactants (1-pentanol, Triton X-100, 2-propanol, and methanol) were tested. We found that at low surfactant concentrations, the SHS longevity in surfactant solutions was larger than that in pure water and increased with increasing surfactant concentrations. This enhancement was attributed to the accumulation of surfactant molecules at the gas–liquid interface, which caused a barrier effect and reduced the liquid-side mass transfer coefficient. At high surfactant concentrations, however, the SHS longevity decreased sharply due to a sudden wetting transition, probably driven by surface energy minimization. Overall, our results underscore the importance of accounting for surfactants when estimating the SHS longevity in real-world applications. Extending the longevity of gas on underwater SHS is crucial for applications, such as drag reduction, antibiofouling, and anticorrosion. Our findings suggest a novel approach to enhance the SHS longevity by introducing small amounts of surfactants into the liquid environment. Furthermore, this work provides a potential framework for quantifying the barrier effect of surfactants on interfacial mass transfer under static conditions, with significant implications for the chemical processing and wastewater treatment industries.

Future studies are needed to investigate the effect of surfactants on the SHS longevity under flow conditions and on nanoscale textures, as well as to examine how surfactant molecular structure affects SHS longevity. Future efforts should also aim to elucidate the mechanisms underlying the extended or reduced SHS longevity due to the surfactant effects, for example, by employing confocal microscopy to visualize the time evolution of the gas–liquid interface during the wetting process and by using numerical simulations to model the diffusion process and the barrier effect of surfactants.

## Supplementary Material



## Data Availability

The original contributions presented in this study are included in the article/Supporting Information. Further inquiries can be directed to the corresponding author(s).

## Notes

Additional experimental results and data analysis including time-series images showing the wetting transition, one-dimensional gas diffusion model, comparison of measured static contact angle to models, mass transfer coefficient as a function normalized surfactant concentration, and a comparison of measured mass transfer coefficient to Sardeing’s model (PDF)
