# Supplementary material for: Effect of Surfactants on Longevity of Submerged Superhydrophobic Surfaces
Source: Langmuir. 2025 Dec 24;42(1):1789–99. doi: 10.1021/acs.langmuir.5c05968 (PMC12836326; doi:10.1021/acs.langmuir.5c05968)
Supplement: Supplementary file 1 [file la5c05968_si_001.pdf]

Supporting Information for

Effect of surfactants on longevity of submerged superhydrophobic surfaces

Ankit Gupta, Hangjian Ling*

Department of Mechanical Engineering, University of Massachusetts Dartmouth, Dartmouth, MA 02747, USA

One dimensional gas diffusion model

Supplementary Figure S1

Supplementary Figure S2

Supplementary Figure S3

Supplementary Figure S4

*Corresponding author. E-mail address: hling1@umassd.edu (H. Ling)

One-dimensional gas diffusion model

We proposed a one-dimensional (1D) gas diffusion model to estimate the longevity of superhydrophobic surface (SHS). In this model, a thin layer of gas with height h_{1D} was exposed to an undersaturated liquid with a semi-infinite domain. The initial gas concentration in the liquid was prescribed as c_∞, and the gas concentration at the interface was fixed as a constant c_i ($c_i > c_\infty$). The diffusion of gas from the thin gas layer to the liquid was governed by the following equation:

$$\frac{\partial c}{\partial t} = D_g \frac{\partial^2 c}{\partial y^2}, \tag{1}$$

where c is the gas concentration in the liquid, D_g is the diffusion coefficient of gas in the liquid (here, for the diffusion of air in water at room temperature, D_g=2.0 ×10^{-9} m^2/s), t is time, and y is the vertical distance from the gas-liquid interface (y=0 is the location of gas-liquid interface). Assuming the interface was fixed at y=0, Equation (1) has a well-known solution as:

$$\frac{c - c_\infty}{c_i - c_\infty} = 1 - erf\left(\frac{y}{2\sqrt{D_g t}}\right). \tag{2}$$

According to Fick's first law, the mass flux at the gas-liquid interface can be calculated as:

$$J = D_g \frac{\partial c}{\partial y}|_{y=0} = \frac{(c_i - c_\infty)}{2}\sqrt{D_g/\pi t}. \tag{3}$$

Therefore, the time when all the mass within the thin layer to be dissolved into the liquid can be found as:

$$t_f = \frac{\pi}{4}\frac{\rho_g{}^2 h_{1D}^2}{D_g (c_i - c_\infty)^2}, \tag{4}$$

where ρ_g is the density of gas (here for air, ρ_g=1.2 kg/m^3). Considering that a SHS with texture height of h and gas fraction of ϕ_g has the same amount of gas to the 1D gas layer with thickness of $h_{1D}=h\phi_g$, we estimated the time for all gas on the SHS to be dissolved as:

$$t_f = \frac{\pi}{4}\frac{\rho_g{}^2 h^2 \phi_g^2}{D_g (c_i - c_\infty)^2}. \tag{5}$$

It should be noted that Equation (5) is same to the SHS longevity model proposed in our early work [1] based on two-dimensional gas diffusion simulations.

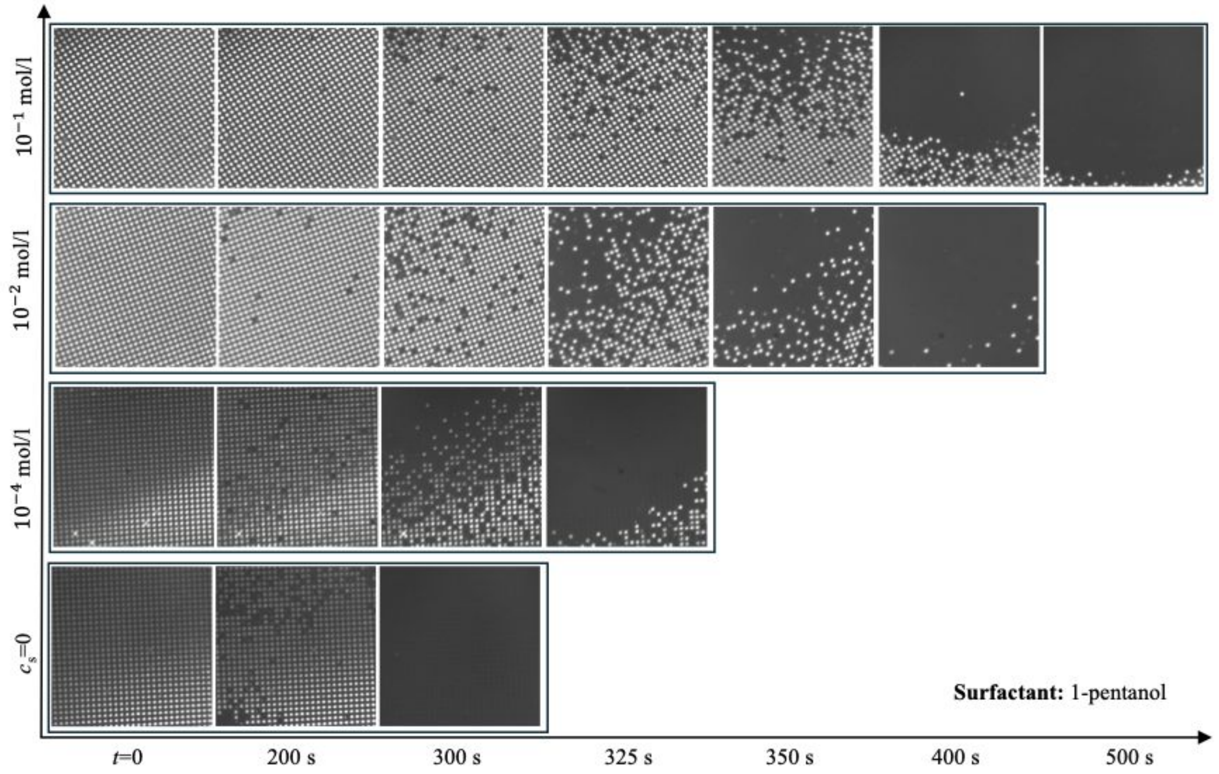

Supplementary Figure S1. Time-series images showing the wetting transition for SHS with micro-holes in pure water and surfactant solutions.

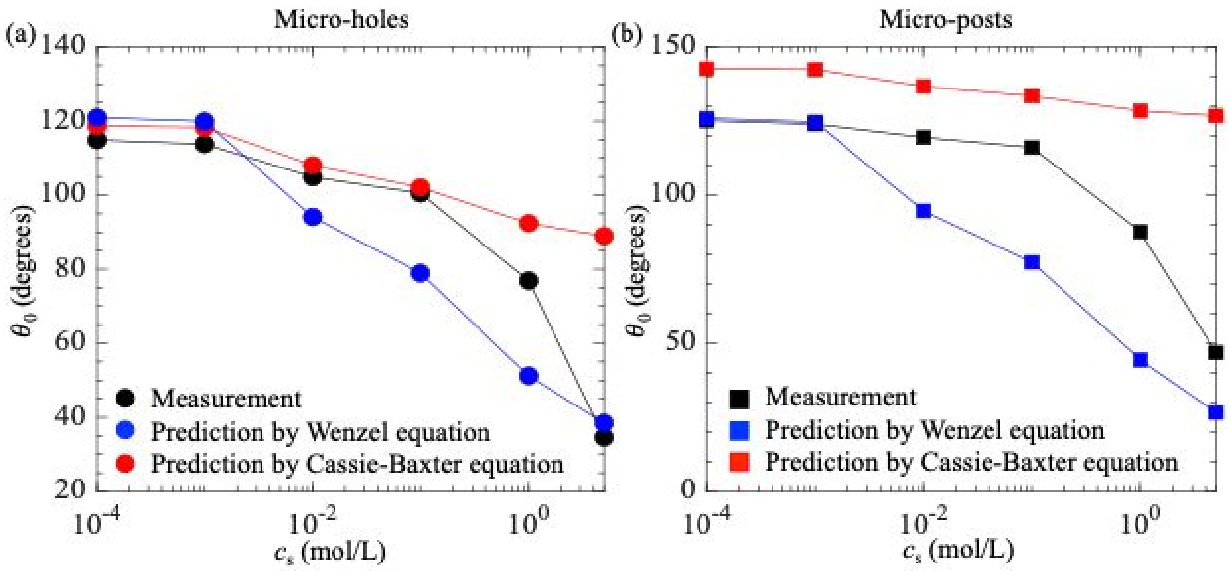

Supplementary Figure S2. Measured static contact angle and predicted static contact angle based on Wenzel equation and Cassie-Baxter equation for SHS with (a) micro-holes and (b) micro-posts.

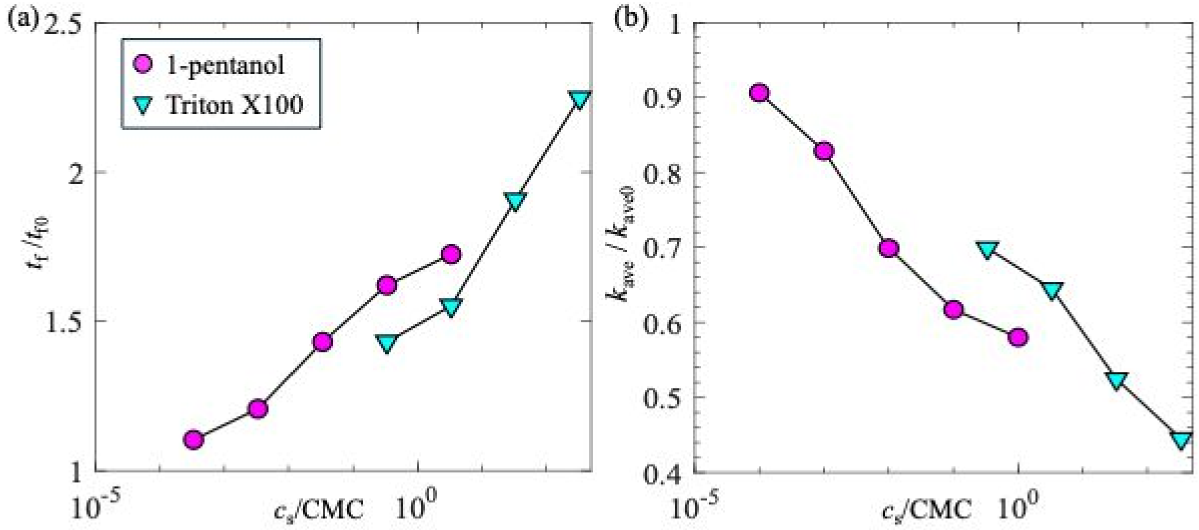

Supplementary Figure S3. (a) SHS longevity and (b) averaged mass transfer coefficient as a function normalized surfactant concentration by CMC. The results are based on SHS with micro-holes. Results for methanol and 2-propanol are not shown since they do not have a CMC value.

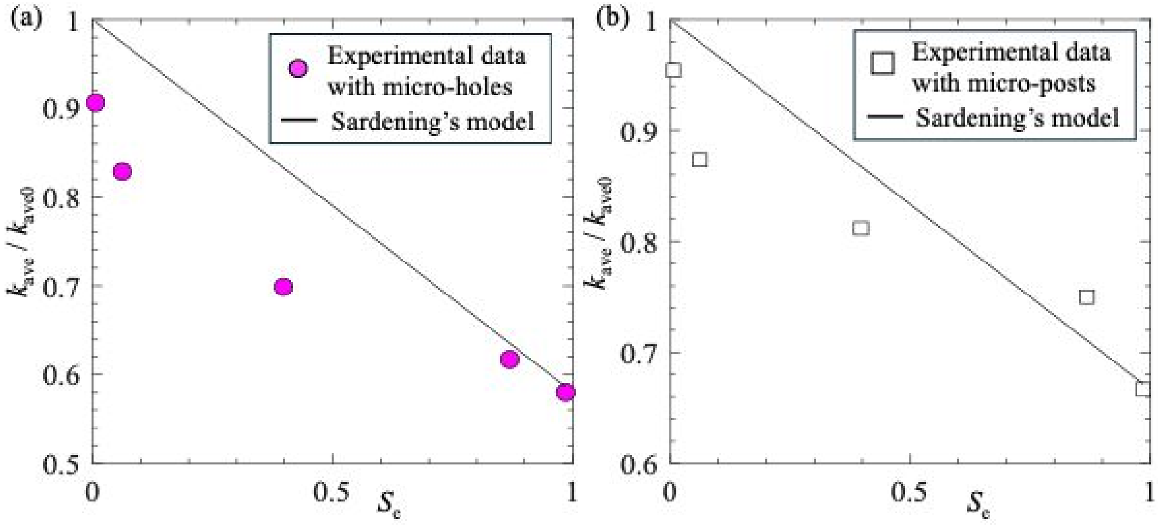

Supplementary Figure S4. (a-b) A comparison of experimental data to the Sardening's model for the mass transfer coefficient as a function of surface coverage ratio (S_e) by surfactant. The experimental data are for 1-pentanol solutions.

Reference

1. Bourgoun A, Ling H. 2022 A General Model for the Longevity of Super-Hydrophobic Surfaces in Under-Saturated, Stationary Liquid. *J Heat Transfer* 144, 042101. (doi:10.1115/1.4053678)
